# The role of genome and gene regulatory network canalization in the evolution of multi-trait polymorphisms and sympatric speciation

**DOI:** 10.1186/1471-2148-9-159

**Published:** 2009-07-09

**Authors:** Kirsten HWJ ten Tusscher, Paulien Hogeweg

**Affiliations:** 1 Department of Scientific Computing, Simula Research Laboratory, P.O. Box 134,1325 Lysaker, Norway; 2 Theoretical Biology and Bioinformatics Group, Utrecht University, Padualaan 8, 3584 CH Utrecht, The Netherlands

## Abstract

**Background:**

Sexual reproduction has classically been considered as a barrier to the buildup of discrete phenotypic differentiation. This notion has been confirmed by models of sympatric speciation in which a fixed genetic architecture and a linear genotype phenotype mapping were assumed. In this paper we study the influence of a flexible genetic architecture and non-linear genotype phenotype map on differentiation under sexual reproduction.

We use an individual based model in which organisms have a genome containing genes and transcription factor binding sites. Mutations involve single genes or binding sites or stretches of genome. The genome codes for a regulatory network that determines the gene expression pattern and hence the phenotype of the organism, resulting in a non-linear genotype phenotype map. The organisms compete in a multi-niche environment, imposing selection for phenotypic differentiation.

**Results:**

We find as a generic outcome the evolution of discrete clusters of organisms adapted to different niches, despite random mating. Organisms from different clusters are distinct on the genotypic, the network and the phenotypic level. However, the genome and network differences are constrained to a subset of the genome locations, a process we call genotypic canalization. We demonstrate how this canalization leads to an increased robustness to recombination and increasing hybrid fitness. Finally, in case of assortative mating, we explain how this canalization increases the effectiveness of assortativeness.

**Conclusion:**

We conclude that in case of a flexible genetic architecture and a non-linear genotype phenotype mapping, sexual reproduction does not constrain phenotypic differentiation, but instead constrains the genotypic differences underlying it. We hypothesize that, as genotypic canalization enables differentiation despite random mating and increases the effectiveness of assortative mating, sympatric speciation is more likely than is commonly suggested.

## Background

Disruptive selection is a major force in driving the formation of biological diversity. The diversity it results in may be sexual dimorphism, within species polymorphisms or speciation [1]. Disruptive selection can be caused by a variety of ecological processes, such as resource competition, frequency dependent predation, sexual conflict and multiple-niche environments [1,2]. Adaptive radiations, such as the ones leading to the diverse species flocks of cichlids and anolis lizards are perhaps the most impressive examples of biological diversification processes.

In asexual species disruptive selection easily leads to clonal species formation. However, in sexually reproducing species recombination between parental genomes has classically been considered to constrain the buildup of discrete differences. The idea is that if any divergence occurs, mating between different parents will lead to hybrid offspring that are an unfit amalgam of the diverged parental properties, thus destroying the built up divergence. This view has been confirmed by various modelling studies. In population genetic model studies of bacteria it was shown that phenotypically distinct clusters can only evolve if the recombination mutation ratio was below a certain threshold [3,4]. Similarly, in classical models of obligate sexual organisms it has been demonstrated that evolution of distinct phenotypic divergence requires the evolution of assortative mating leading to sympatric speciation [5-9] (an exception being [1,10]).

There are 2 problems with the predictions flowing from these models. The first problem is the occurrence of species with multi-trait, genetically based polymorphisms. Well known examples are insects with mimicry such as butterflies [11-13], but also species such as cuckoos, grove snails, lady beetles, moths, and ants. These genetic polymorphisms demonstrate that apart from assortative mating other mechanisms must exist that allow for phenotypic divergence under sexual reproduction. Another problem is that extensions of the above discussed model studies have shown that if assortativeness is costly [1,7,14], if mate choice is based on a character unrelated to the ecological character under disruptive selection [5,15], or if observation of mate phenotype is noisy [16], that is, if assortativeness is modelled more realistically, assortativeness is very hard to evolve. However, there is increasing evidence for the evolution of assortative mating and it's involvement in sympatric speciation at least in particular situations [17-20].

If we compare the above modelling studies some key general properties can be found. First, only point mutations changing the allele type at a certain gene locus are being considered in these models. Second, the genetic architecture is assumed to be simple and constant. Third, the mapping from genotype to phenotype is assumed to be linear.

Over the last two decades it has become increasingly clear that mutations such as duplication, deletion and divergence of genes, regulatory elements and even large chromosomal stretches play a comparable or even larger role in adaptive evolution than the classically studied changes in allele types [21-26]. The resulting differences in gene copy numbers, genome architecture and gene regulatory network organization have been found to contribute to interspecies differences [27-31], and within species polymorphisms [27,28,32-36]. For example, some genetic polymorphisms are caused by a supergene, a cluster of closely linked genes responsible for generating the morph specific characteristics [12,13,37]. Furthermore, numerous modelling studies have shown the importance of a non-linear genotype phenotype mappings for the flexibility of the evolutionary dynamics [38,43].

Therefore, in this study we will explicitly investigate the influence of a flexible, evolving genome and gene regulatory network architecture and the resulting non-linear genotype phenotype mapping on the evolution of phenotypic divergence under sexual reproduction. We use an individual based model in which organisms compete in a multi-niche environment to induce disruptive selection. As such, our model is an extension of the approach used by both Kondrashov and Gavrilets [6,8,9], who studied a 2 niche situation, but is substantially different from the models used by Doebeli and co-workers [5,7], where a single optimal niche is used and differentiation requires deviation from this optimal niche. Organisms have a genome consisting of genes and transcription factor binding sites. Mutations affect individual genes or binding sites or stretches of genome. The genome codes for a gene regulatory network that determines the gene expression dynamics and hence the phenotype of the individual. The model thus contains a complex, non-linear, many-to-one genotype phenotype mapping. For comparison purposes we use a null model in which individuals have a simple genome only containing genes, and in which mutations only affect whether a gene is expressed or not. The null model thus has a linear, one-to-one genotype phenotype mapping.

Comparison of the full and null model allows us to pinpoint the effects of a flexible genetic architecture and non-linear genotype phenotype mapping on phenotypic divergence under sexual reproduction.

We show that the presence of a flexible genetic architecture combined with a non-linear genotype-phenotype map allows the evolutionary process to go to a part of the mapping where limited genotypic differences are sufficient to generate phenotypic differentiation. This process enables phenotypic divergence under random mating by reducing the impact of recombination and increasing hybrid fitness. In addition, it enhances phenotypic divergence under assortative mating. We suggest that both the potential for discrete phenotypic variation despite random mating and the larger effectiveness of assortative mating may increase the likelihood of evolving assortative mating and sympatric speciation.

## Methods

Table 1 describes default parameter values used for the simulations described in this article. In addition, alternative parameter settings used for the additional simulations described in Additional file 1 are listed.

**Table 1:** Parameter settings of the model.

parameter	default value	alternative values
grid size	60 by 140	-
initial nr organisms	1500	-
total evolutionary time	500000	-
death rate	0.1	-
nr different niches	20	2/7/14
nr of types of TF genes	4	-
nr of types of phenotype genes	20	-
nr of network iterations	10	30/60
initial network connectivity	2	-
*α*	0.8	0.4
HD_*th*_	6	only nearest
*β*	250	25/100
*γ*	1.	0.6
tfbs duplication rate	0.0001	0.001/0.00001
tfbs deletion rate	0.000165	0.00165/0.0000165
tfbs innovation rate	0.00005	0.0005/0.000005
tfbs type change rate	0.00005	0.0005/0.000005
tfbs weight switch rate	0.00005	0.0005/0.000005
gene duplication rate	0.0003	0.003/0.00003
gene deletion rate	0.00045	0.0045/0.000045
macromutation rate	0.005	0.05/0.0005
gene state mutation rate	0.003	-
assortativeness mutation rate	0.075	0.0075
assortativeness mutation std.	0.15	-
reproduction radius	2	1/4/global
competition radius	2	1/4/global

The table shows the default parameter settings we used for the simulations described in the article. In addition, alternative parameter values used for the additional simulations described in Additional file 1 are shown. The number of network iterations refers to the maximum number of times the state of the gene regulatory network is updated starting from the birth state of the organism to determine it's phenotype. TFBS mutation rates are per TFBS, gene mutation rates are per gene. Macromutations, assortativeness mutations and TFBS innovation rates are per genome. The ratios between the different mutation rates where chosen such that macromutations, which occur per genome, have a rate that is an order of magnitude larger than that of TFBS and gene mutations, which occur per TFBS or gene. To prevent either gene evolution or TFBS evolution from dominating the dynamics of our model, we aim for similar overall gene and TFBS mutation rates. Therefore, as a single gene usually has multiple TFBS, and more types of mutations occur on TFBS than on genes, TFBS mutation rates are kept somewhat lower than gene mutation rates. Finally, to prevent excessive genome growth, TFBS and gene deletion rates are chosen to be approximately 1.5 times larger than their duplication rates.

### Full model

#### Individual-based spatial model

**Figure 1:**
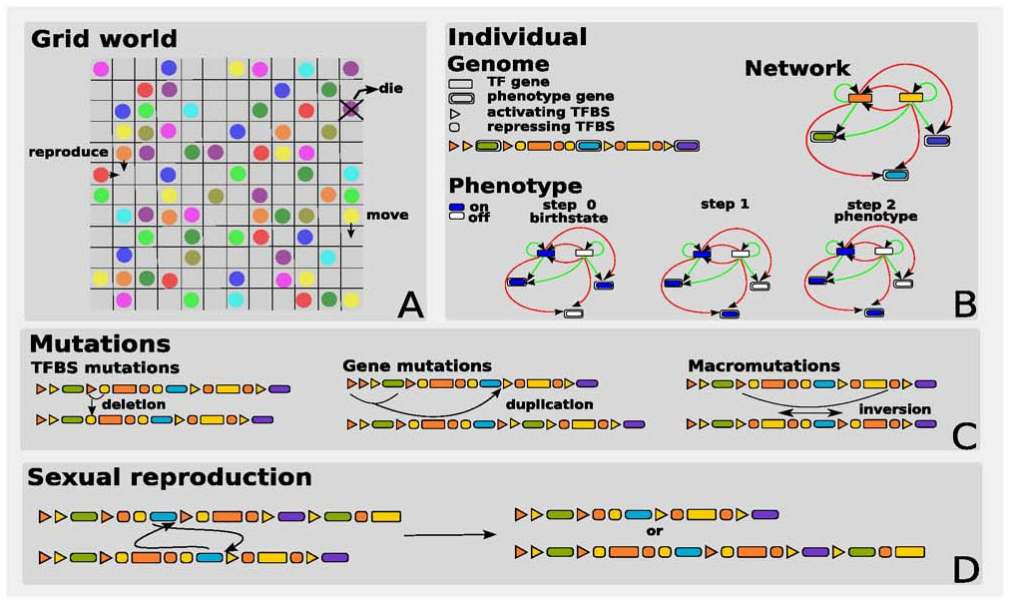
**Overview of the model**. **A **Organisms reside on a 2D grid world in which they live, move, reproduce and die. **B **Individuals contain a genome which consists of a linear array of genes and TFBS upstream of these genes. There are two types of genes, transcription factor (TF) and phenotype genes. The genome codes for a gene regulatory network, with genes as nodes and TFBS as edges of the network. The edges represent the activating (green) or repressing (red) influence of TF genes on the expression of other genes. A birth state dictates the initial expression state of the genes. The network edges determine how gene states are updated as a function of the state of other genes. The phenotype of the individual is determined by the final state of only the phenotype genes. This final state is reached once updating leads to a steady state gene expression pattern or if a maximum number of update steps have been performed. **C **Mutation events that can occur involve either individual TFBS, individual genes, or stretches of genome. **D **Sexual reproduction is implemented as a crossing over between two parental genomes to create offspring genomes. Crossing over occurs between homologous locations: where the two parental genomes have the same gene type.

We use an individual-based model of a population of *in silico *organisms living on a two-dimensional grid world (Figure 1A, typically 60 × 140). Each position of the grid can be either empty or occupied by a single individual. At the start of the simulation the population is initialized with 1500 identical individuals that are placed randomly in the middle region of the grid. Organisms within a certain radius (default 2) of an empty position compete to reproduce into that position using probabilistic fitness-based selection.

Furthermore, organisms move by random diffusion [44] and die with a constant probability (default 0.1). The population is allowed to evolve for 500000 time steps.

We use a spatial model as we believe this more closely reflects the locality and discreteness of populations of interacting organisms. In addition, local interactions are more computationally efficient than all against all interactions. However, our results do not depend critically on this model feature (see Additional file 1).

#### Genes, Genome, Gene Regulatory Network and Phenotype

Organisms have a linear genome that contains genes and their upstream regulatory regions composed of transcription factor binding sites (TFBSs) (Figure 1B). There are two types of genes: transcription factors (TF), which influence the expression of genes, and phenotype genes, which determine the phenotype of the organism. We have 4 types of TFs, and hence also 4 types of TFBSs to which TFs bind. In addition, we use 20 types of phenotype genes. At the start of the simulation, the genome of the first 1500 identical individuals is generated by randomly ordering single copies of the 24 gene types and randomly placing an average of 2 TFBSs upstream of each gene.

If TFs are expressed they bind to the corresponding TFBSs, thus exerting an influence on the genes that have these TFBSs in their upstream region. The genome thus codes for a gene regulatory network, in which vertices correspond to genes and edges are determined by the TFBSs upstream of each gene (Figure 1B). To keep our model computationally tractable, we use a Boolean gene network in which genes are either expressed (1 or *on*) or not expressed (0 or *off*). The next state of a gene *i *depends on its current state and the occupancy of its TFBSs according to the following expression [45,46]:(1)

with  the expression state of gene *i *at time *t *and *w*_*ij *_the weight or impact of the TFBS for TF *j*.

Weights can be +1 or -1, determining whether a TF enhances or suppresses gene expression. Updating is performed synchronously for all genes. The advantage of this function is that it is invariant with the number of TFBSs per gene, which is not the case for the frequently used boolean updating functions. In our model, we take the phenotype of an organism as the (final) expression pattern of the 20 phenotype genes, ignoring the state of the TF genes (Figure 1B). To determine the phenotype of an organism, its gene network is initialized with a so-called birth state. Typically, a single gene expression pattern generated at the start of the simulation is used for all individuals as the birth state. The birth state consists of all TF genes switched *on *and a randomly drawn state for the 20 phenotype genes. After this, the network is updated until it converges or 10 network update steps have been performed. The final state often corresponds to a fixed point attractor of the network. However, it may also be a state of a cyclic attractor, or a transient state.

We define the phenotype of an organism as the *on/off *expression pattern of the 20 phenotype genes (a bit pattern of length 20). If multiple copies of a certain phenotype gene are present, the gene type is considered *off *if all copies are *off *and *on *if at least one is *on*. Note that, because of the fixed birth state, the fixed number of update steps and the deterministic update rule, the phenotype of an organism follows deterministically from the genotype.

#### Mutations

Mutations occur at reproduction on the newly produced genome (Figure 1C, Table 1). From small to large: TFBS mutations may change which type of TF binds, or the weight of the TFBS (i.e. activating or repressing). In addition, a TFBS may be duplicated, deleted or innovated. Innovations add a TFBS of any possible type, whereas duplications copy and add a TFBS of a currently present type. Innovation thus allows for the reinvention of lost TFBS types. Single genes, including their upstream regions of TFBSs, may be duplicated or deleted. Finally, macromutations occur on large segments of genome and may lead to duplication, deletion or inversion of the segment.

#### Disruptive selection: multiple niches and competition

To impose frequency-dependent disruptive selection we use a multiple-niche environment and local, frequency-dependent competition. We introduce 20 different ecological niches to which individuals can adapt. All these niches are present at each grid position, as we want to study sympatric and not parapatric divergence, and can thus be interpreted as ecological roles rather than localized, physical habitats. Niches are defined as specific phenotypes, that is, a specific expression pattern of the phenotype genes that is needed to be optimally adapted to a particular niche, similar to the approach in [47]. At the start of a simulation, we randomly pick 20 different niches from the space of 2^20 ^possible niches.

Competition occurs between individuals within a certain radius of one another (default 2). The strength of competition depends on the similarity between competing individuals and the number of competing individuals.

The fitness *f *of an organism *i *is defined as:(2)

with *a *reflecting how well adapted an individual is to the different niches, and *c *reflecting the competition an individual experiences from its local neighbors.(3)(4)

Total adaptedness *a *is summed over all niches (*n *∈ *niche*) in the environment. Adaptedness of an individual *i *to a single niche *n *(*g*(*i*, *n*)) decreases as a function of the Hamming Distance (HD) between the phenotype of organism *i *and the ideal phenotype for niche *n*. Parameter *α *determines how strongly adaptedness decreases with increasing *HD*_*in*_. Only niches to which the organism has a distance less than *HD*_*th *_contribute to overall adaptedness (default *HD*_*th *_= 6). Total competition *c *is summed over the competition individual *i *experiences from all neighboring individuals present (*j *∈ *nb*) and is normalized for the maximum number of neighboring individuals (*nb*_*max *_= 24 for a competition radius of 2). Competition between individual *i *and *j *decreases as a function of the distance *HD*_*ij *_between the individuals. Parameter *γ *determines how strongly competition decreases with *HD*_*ij*_. Parameter *β *determines the maximum strength of competition. The maximum function ensures that the minimum of the competition function is 1/*β*.

To prevent excessive increase of genome length during evolution, a fitness penalty is added. Typically we use *HD*_*in *_= *HD*_*in *_+ *max*((*nrg *- 55)*mod*20, 0), with *nrg *the number or genes. This increases the effective HD to a niche used to determine an individuals fitness.

Note that during reproduction we compare the fitness *f *of all individuals *i *in a neighborhood radius around the empty spot in which an offspring individual can be placed. In contrast, when computing the competition *c *experienced by an individual *i *we use a neighborhood radius around the individual *i *itself.

#### Modes of Reproduction

We use 3 different modes of reproduction in our study.

First, we use sexual reproduction with random mating. Given an empty position on the grid, we use probabilistic fitness-based selection to choose (without replacement) two parents from the local neighborhood *nb *(radius 2 around the empty position). Explicitly, an organisms chances of becoming a parent are proportional to it's local relative fitness:(5)

Second, we use assortative sexual reproduction. The first parent is selected according to Eq. 5. The chances for the remaining local individuals to be chosen as the second parent are the product of Eq. 5 and the mate choice preference of the first parent (*mc*, see section Assortative Mating). Per mating, each local individual can be first or second parent, but not both. There are no explicit costs of being choosy in terms of the first parent not mating if it does not find a similar enough partner [14]. However, if there is positive assortativeness, individuals with rare phenotypes will be chosen less often to mate with, resulting in implicit costs of assortativeness.

Finally, as a control case, we use asexual reproduction. Again, we use probabilistic fitness-based selection as defined in Eq 5 to choose a single parent.

#### Sexual Reproduction

Sexual reproduction is implemented as a homologous crossing-over event between the genomes of two parents. We randomly choose a gene from parent *i*, and we match it with a random gene *of the same type *in parent *j*. Note that there may be multiple copies of this gene type in parent *j *to choose from. Crossing over is then performed at the two selected gene positions. With equal probability one of the two potentially resulting genomes is used to form the offsprings genome (Figure 1D). After recombination, mutations occur on the newly formed genome.

Note that offspring may arise which do not contain at least one copy of each gene type in their genome. Such offspring are infertile and assigned a fitness *f *= 0.

#### Assortative Mating

If we allow for assortative mating, organisms have an extra parameter, *a*, determining their level of assortativeness. At the start of a simulation we initialize *a *at zero. Assortativeness influences the likelihood of a mating between two individuals *i *and *j *as follows:(6)

where *HD*_*ij *_is the Hamming distance between the phenotypes of individuals *i *and *j*, and *HD*_*max *_= 20 (the number of phenotype genes). The parameter *a *mutates at a rate of 0.075, and the mutation size is drawn from a Gaussian distribution (*μ *= 0, *σ *= 0.15). Note that the mutation rate and mutation size are relatively large and that using a single parameter value to determine choosiness is rather simplistic. These choices were made mainly to increase computational efficiency. Note that the generality of the obtained results was tested in additional simulations with lower mutation rates or where assortativeness was encoded in 6 additional genes rather than a separate parameter (see Additional file 1).

### Null model

For comparison purposes we also constructed a null model. The null model, like the full model, is an individual based spatial model in which organisms move, reproduce and die. In addition, in the null model we have a multiple-niche environment, 3 modes of reproduction, homologous recombination of parental genomes under sexual reproduction and the same implementation of assortative mating as in the full model. The differences between the null and the full model are in the genome, mutations and the genotype-phenotype mapping. In the null model, organisms have a genome with 20 phenotype genes. The organisms lack TFs and TFBSs, so there is no gene regulatory network. In addition, genome architecture is fixed, and mutations only occur on gene expression state, toggling from *on *to *off *and vice versa. As a consequence, the genome codes directly and linearly for the phenotype.

### Analysis

#### Genome alignment

To study genome order, we perform genome alignments using the multiple sequence alignment program Clustalw, version 1.83 (see http://www.clustal.org and [48]). The program first performs pairwise alignments between all pairs of sequences, lining up the sequences side by side to identify the regions of similarity and difference between sequences. Next, a hierarchical clustering based on these sequence similarities is used to construct a similarity tree. Finally, sequential pairwise alignments along this tree are performed to obtain an overall multiple sequence alignment. Default Clustalw settings are used, except for gaps and scoring matches. We use a gap opening penalty score of 1 and a gap extension penalty score of 0.1, such that gaps can be introduced at low cost, maximizing the detection of gene types at similar positions. We use the identity matrix for scoring matches, thus treating all gene types as equally different from one another. After an alignment, an additional processing step is performed. Alignment positions for which a fraction > 0.95 of the individuals have a gap are removed from the alignment. This makes alignments visually better interpretable.

#### Phenotype clustering

We cluster individuals on phenotypic similarity using R, a programming language and software environment for statistical computing and graphics, version 2.7.2 (see http://www.r-project.org and [49]).

We apply hierarchical clustering with complete linkage and Manhattan distances (these correspond to phenotypic HD).

#### Terminology

Below we define a number of terms used in the rest of the article.

### Individuals

**Niche (hamming) distance**: number of phenotype genes for which the expression state differs from the ideal niche expression state.

**Phenotype (hamming) distance**: number of phenotype genes for which a pair of individuals have a different expression state.

**Individuals adapted to the same niche**: individuals that have the shortest hamming distance to the same niche.

**Minimum hamming distance**: hamming distance of an individual to the nearest niche.

**Hybrid**: offspring of two parents that have specialized on different niches.

**Non-hybrid**: offspring of two parents that have specialized on the same niche.

### Groups of individuals

**Morph**: a group of individuals adapted to the same niche that is not reproductively isolated from individuals adapted to different niches (i.e. under random mating).

**Species: **a group of individuals adapted to the same niche that are (largely) reproductively isolated from individuals adapted to other niches (i.e. under significant levels of assortative mating)

**Morph/species heterogeneity**: the average of pairwise phenotype hamming distances between all individuals in a morph/species. This measures the phenotypic diversity of individuals adapted to the same niche.

**Morph/species cohesion**: the ratio of average between morph/species hamming distances (average HD between all pairs of individuals belonging to different morphs/species) and within morph/species hamming distances (average HD between all pairs of individuals belonging to the same morph/species). This measures the quality of phenotypic clustering of individuals adapted to the same niche.

### Population

**Phenotypic distance**: average of pairwise phenotype hamming distances between all individuals in the population normalized over the number of phenotype genes. The maximum normalized distance between two individuals is one.

**Genome distance**. To efficiently compute the evolution of genome distance over time, we use an abstract representation of genome order suitable for comparing genome order of individuals without the need for alignment. This representation scores per gene type how often it is followed on the genome by a gene of another type, resulting in a row of 24 × 24 numbers that represents local but not global genome order. Genome distance is computed as the average pairwise hamming distance between the thus represented genome orders of all individuals in the population. Note that, the maximum distance between two individuals with completely differently ordered genomes would be two times the number of genes they have in their genome. We therefore normalize genome distances over two times the average number of genes per individual in the population. This results in a maximum normalized genome distance between two individuals of 1.

**Network distance**. Similarly, to efficiently compute evolution of network distance, we score per gene type for each possible TFBS type whether it is present in the upstream region of that gene (1/-1 present, 0 absent) and whether it activates or represses the expression of that gene (1 activates, -1 represses). If multiple copies of a gene type are present results are averaged, so non integer scores are possible. This results in a row of 24 × 4 numbers representing an individuals network architecture. Network distance is computed as the average pairwise hamming distance between the thus represented network architectures of all individuals in the population. These hamming distances are normalized over the average number of genes per individual in the population × the number of TFBS types (4) (gives the maximum number of differences) × 2 (maximum size of a single difference), which equals the maximum distance between two networks. The maximum normalized network distance between two individuals thus is 1.

## Results

Next, we will discuss in detail the results of a few typical runs using the default parameter settings described in Table 1. After this, the generality of these results will be discussed.

### Phenotypic diversity under random mating

#### Phenotypic divergence

**Figure 2:**
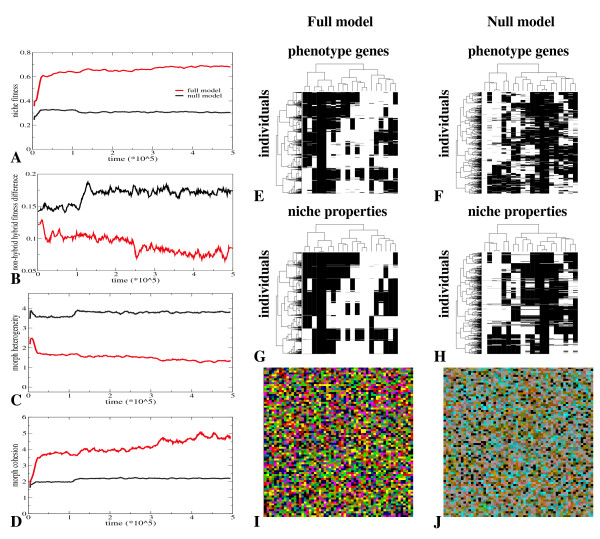
**Evolution of phenotypic diversity under random mating in the full and the null model**. For an explanation of terms and measures see Methods. **A **Evolution of niche-fitness (equation 2) in the full and the null model. **B **Evolution of the difference in niche-fitness between hybrids and non-hybrids in the full and the null model. **C **Evolution of morph heterogeneity in the full and the null model. **D **Evolution of morph cohesion in the full and the null model. **E **and **F **Distribution of phenotypes present in the population at time 500000, for the full (E) and null (F) model. A random 10% sample of individuals are taken from the population. Phenotypes of individuals are determined by the state of their 20 phenotype genes (see Methods). Black: gene not expressed, white: gene expressed. Individuals (rows) are clustered based on phenotype similarity, phenotype genes (columns) are clustered based on across population expression pattern similarity. **G **and **H **Distribution of niches on which the population has specialized at time 500000, for the full (G) and null (H) model. The same 10% sample of individuals as in E and F are shown. Niches to which individuals have the lowest HD are shown. A niche is characterized by the phenotype gene expression pattern needed to be perfectly adapted to that niche (see Methods). Black: gene that should be off in this niche; white: gene that should be on in this niche. Note that for comparison purposes individuals (rows) and niche properties (columns) are clustered the same as in E and F, respectively. **I **and **J **Snapshots of the distribution of individuals on a 25% part of the grid world at time 500000 in the full (I) and null (J) model. Black: empty spot; grey: organisms with *HD *> 3 to their nearest niche; colors: organisms with *HD *< = 3 to their nearest niche, with different colors indicating different nearest niches.

In the full model, but not in the null model, we find the evolution of discrete phenotypic diversity under random mating. In Figure 2 we show a comparison of the evolutionary outcome in the full and null model. In the full model we see a substantial increase in niche fitness over evolutionary time up to a level of 0.7 (Figure 2A). In addition, we see that fitness differences between hybrids (offspring from parents adapted to different niches) and non-hybrids (offspring from parents adapted to the same niche) are small and decrease over evolutionary time (Figure 2B, 0.115 at begin, 0.073 at end of evolution). Furthermore, we observe a decrease in the heterogeneity (Figure 2C) and an increase in the cohesion of the different evolved morphs (Figure 2D). Indeed, in Figure 2E we see that at time 500000, a total of 8 clearly distinct and rather homogeneous phenotypic clusters have evolved. From a comparison with Figure 2F we see that individuals from the same phenotypic cluster have specialized on the same niche. In this particular simulation, organisms have specialized on 8 of the 20 present niches: On average we found divergence into 8.625 phenotypic clusters (n = 17, std = 1.857). In Figure 2G we show the spatial distribution of individuals. Individuals with a *HD *> 3 to the nearest niche are colored grey, others are colored based on the niche they have specialized on. We see that most individuals are well adapted to a particular niche, and we see no apparent spatial clustering of individuals adapted to the same niche. This indicates that the evolution of phenotypic divergence occurs not only despite fully random mating but also in full sympatry.

These findings contrast sharply with our results for the null model, where no evolution of distinct phenotypic clusters occurs (Figure 2F). Instead, the population is polymorphic at all phenotypic loci (all phenotype genes (columns) occur both in expressed (white) and non-expressed (black) state in the population), but the state of the different phenotypic loci remains largely uncorrelated. In agreement with this we see that the evolutionary process stagnates and niche fitness remains at a low level of 0.3 (Figure 2A). In addition, fitness differences between hybrid and non-hybrid offspring remain large (Figure 2B). Similarly, morph heterogeneity remains high (Figure 2C) and morph cohesion remains low (Figure 2D). Figure 2J shows the distribution of individuals over space. We see that a large part of the population is not well adapted to any particular niche (grey color), in agreement with the low average fitness values evolved. So, although the evolution of diversity under frequency dependent selection is trivial, the discrepancy between the full and the null model clearly illustrates that it is far less straightforward to evolve discrete phenotypic diversity under random mating. Note that under asexual reproduction, as expected, discrete phenotypic divergence readily evolves in both the full and the null model (results not shown).

From our results it follows that discrete phenotypic differentiation under random mating is possible in the full but not the null model. The principle difference between these models is in the flexibility of the genetic architecture and the nature of the genotype phenotype mapping. In the next sections we analyst how the flexible genetic architecture and the non-linear genotype phenotype mapping of the full model allow for the evolution of phenotypic differentiation despite random mating.

#### Genotypic canalization

Due to random mating and the random spatial distribution of individuals of different types, there is a continuous production of hybrid offspring from the mating of dislike parents. There will thus be a strong selective pressure for increasing the fitness of hybrids relative to the baseline situation in which hybrids are an unfit amalgam of their parents properties. Indeed, in the full model we see that hybrid fitness increases during evolution, whereas this is not the case in the null model (Figure 2B). This implies that, somehow, this selection pressure is able to interact with the flexible genetic architecture and non-linear genotype phenotype mapping of the full model such that hybrid fitness increases and divergence is enabled.

To see exactly what this selection for increased hybrid fitness does we compare the situation for random mating to the situation of asexual reproduction, both in the full model. In the latter case we have the same genetic architecture and genotype phenotype mapping but no sexual reproduction and hence no selection for larger hybrid fitness.

##### Limiting genotypic differentiation

**Figure 3:**
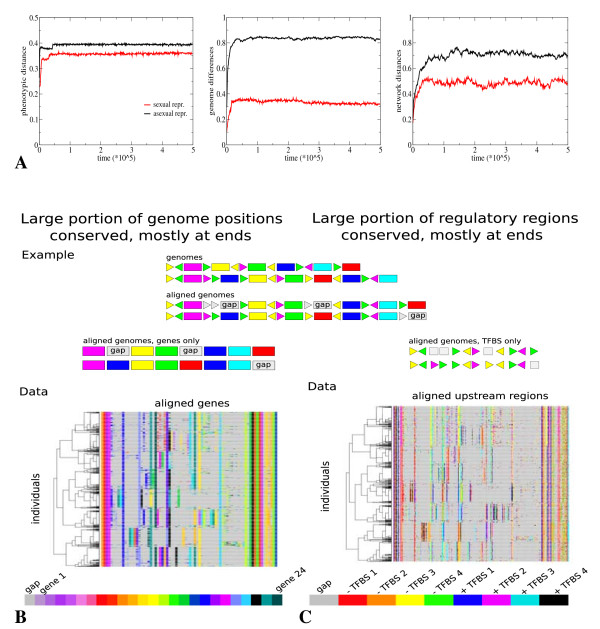
**Genotypic canalization**. **A **Evolution of average, population level phenotypic distances (left), genome distances (middle) and network distances (right) under sexual (red lines) and asexual (black lines) reproduction. For an explanation of the measures used see Methods. **B and C **In the cartoon we show two different, simplified genomes and the genome sequences resulting from a pairwise alignment of these two sequences. Note the introduction of alignment gaps (grey). Next, we show these aligned sequences again if only the genes but not the TFBS (left) are displayed, or if only the upstream regulatory region (URR) with TFBS but not the genes themselves (right) are shown. **B **Data: Aligned genomes with only genes shown. Multiple sequence alignment is performed using Clustalw (see Methods). Genomes shown are for the same individuals as in Figure 2E, and are again clustered based on phenotype. Grey: alignment gaps; color: genes of different types. **C **Data: Aligned genomes with only the URRs with their TFBS shown. Genomes shown are the same as in B and are clustered based on phenotype. We show a maximum of 3 TFBS per gene. Grey: alignment gaps and absent TFBS (if a gene has less than 3 TFBS); color: TFBS of different type and weight.

Figure 3A shows the evolution over time of phenotypic differences (left), genome order differences (middle) and network wiring (right) differences under random mating (red lines) and asexual reproduction (black lines) in the full model (for a definition of the distance measures see Methods, Terminology). Phenotype, genome and network distances are all normalized to lie between zero and one. Note that the dimensionality of the phenotype space (20), genome order space (24 × 24), and network wiring space (24 × 4) is rather different (see Methods), such that comparison of distances in these different spaces is not straightforward. We see that under sexual and asexual reproduction similar phenotypic differences evolve (~0.36 versus ~0.39, factor 1.08 difference), the slightly larger phenotypic differentiation in the asexual situation probably being due to a slightly larger number of different phenotypic clusters (10 versus 8). In contrast, the evolution of genome order and network wiring differences are considerably smaller under sexual compared to asexual reproduction (~0.31 versus ~0.84, factor 2.7 difference, and ~0.48 versus ~0.71, factor 1.5 difference). Not only do these differences evolve more slowly and to lower values, genome order and network wiring differences even decrease slowly over longer evolutionary timescales. We conclude that under (random) sexual reproduction evolution goes to part of the genotype phenotype landscape where genotypic differences underlying phenotypic differentiation are restricted. We will refer to this process as *genotypic canalization *[50,51].

##### Conserving gene types at genome ends

In Figure 3B and 3C we show the genome and network differences evolved at the end of the simulation for the sexually reproducing population. Because of the mutational operators we use in our model, genomes of different individuals can differ in number and ordering of the genes they contain. Therefore, to allow a comparison of genome organization, we performed multiple sequence alignments on the genomes (see Methods). In Figure 3A we show, for the same individuals as shown in Figure 2D, the aligned order of genes on their genomes, ignoring the TFBSs upstream of the genes. Individuals are, as in Figure 2D, clustered based on phenotypic similarity. The different colors indicate genes of the different types. Grey indicates an alignment gap. We see that individuals within the same phenotypic cluster have a very similar genome order. We also see that, although individuals belonging to different phenotypic clusters have a less similar genome order, their genome order is not completely different. For a considerable number of genome positions almost all individuals in the population have the same gene type, irrespective of the phenotypic cluster they belong to. Only for a subset of the genome positions do individuals contain phenotypic cluster specific gene types. Furthermore, we observe that the conserved genomic locations occurs mostly at the two outer ends of the genome, whereas the niche specific genomic locations occur mostly in the middle region of the genome.

##### Conserving TFBS at genome ends

In Figure 3B we show, again for the same individuals, the upstream regulatory regions (URRs, consisting of TFBSs) upstream of the different gene types. The URRs of genes are ordered based on the genome alignment shown in Figure 3A. For each URR we show a maximum of 3 TFBSs, in the rare case that a gene has more TFBSs these are ignored in this Figure. TFBSs are colored based on type (1, 2, 3, 4) and weight (+, -). The grey color indicates the absence of a TFBS, in case a gene contains less than 3 TFBSs in its URR, or an alignment gap. We see that individuals belonging to the same phenotypic clusters have very similar URRs and hence very similar gene regulatory network wiring. We can also see that individuals from different phenotypic clusters have less similar URRs, but that these differences are limited to a subset of the genome locations. A considerable number of genome locations have similar URRs in all individuals, irrespective of the phenotypic cluster the individuals belong to. Furthermore, we observe a clear correlation between the conservation of gene types at certain genome locations (mostly the ends) and the conservation of URRs upstream of those genes.

In contrast to these results, under asexual reproduction, organisms from different morphs differed with respect to gene types and upstream regions along the entire genome length (Figure S1). Quantitative data for the amount of gene type and upstream region conservation for 5 runs under sexual reproduction with random mating and 5 runs under asexual reproduction are given in Additional file 1 (Figure S2).

Our results suggest that, under sexual reproduction, there is a selective advantage of genotypic canalization, predominantly conserving genome ends and restricting differences to the middle genome regions. We conclude that in the full model the selection for increased hybrid fitness gets translated into a selection for genotypic canalization.

#### Non-linear genotype phenotype mapping

The question is how limited genotypic differences can still result in the phenotypic differences needed for adaptation to the different niches.

##### Different usage of conserved gene types

**Figure 4:**
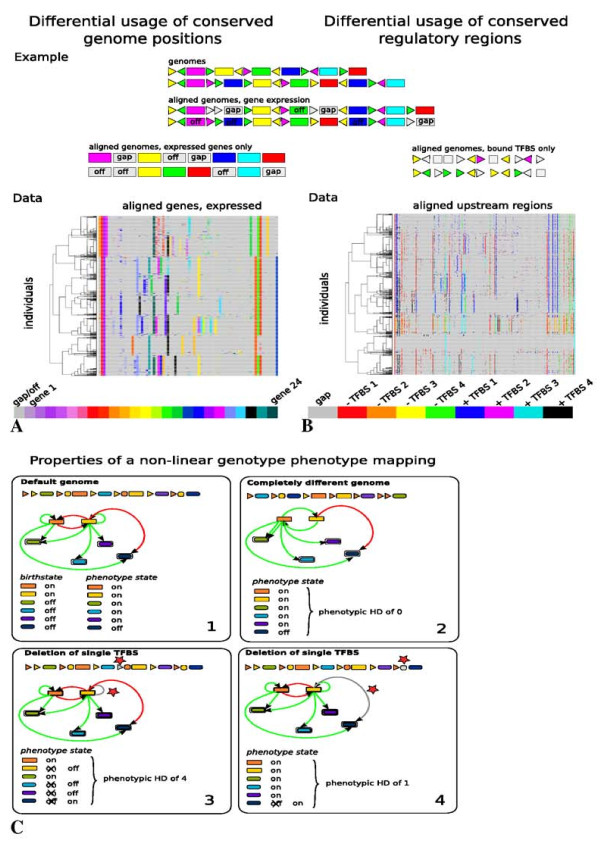
**Phenotypic Diversity under Canalization**. **A and B **As in Figure 3, we show two different, simplified genomes, and the genome sequences we obtain from aligning them. Now we indicate in the aligned genome sequence which genes are not expressed (off), using the same grey color as for alignment gaps. Next, on the left the genes-only aligned sequences are shown if only expressed genes are displayed, and unexpressed genes are given the same grey color as alignment gaps. Likewise, on the right the URR-only aligned sequences are shown if only TFBS bound by a TF (the TF gene is expressed) are displayed, and unbound TFBS are given the same grey color as absent TFBS. **A **Data: Aligned genome order in which only genes that are expressed (on) are shown, non-expressed genes are colored grey. Genomes shown are the same as those in Figure 3B. **B **Data: TFBS present in upstream regions of genes, showing only TFBS that are bound by their corresponding TF. Non-occupied TFBS are colored grey. Genomes and TFBS are the same as those in Figure 3C. **C **Cartoon showing characteristics of the non-linear genotype-phenotype mapping of our model. In 1 we show a default genome, the network it codes for, a gene expression birth-state and the phenotype arising from this birth-state and the network dynamics. In 2, 3 and 4 we show different genomes, coding for different networks, that use this same birth-state as a start state. In 2 we show a very different genome and regulatory network (only 2 of the 8 connections are the same as in 1), resulting in exactly the same phenotype. In 3 and 4 we show genomes that differ from the default genome only by the deletion of a single TFBS (red star), resulting in networks that differ from the default one by missing a single regulatory link (grey line). In 3 this small genotypic change results in a large phenotypic change (4 of the 6 genes change their expression), whereas in 4 this small genotypic change results in a small phenotypic change (only 1 gene changes its expression).

Figure 4A shows the same aligned genomes as in Figure 3A, the only difference being that only expressed genes are shown and unexpressed genes are given the same grey color as alignment gaps. This illustrates that if we look at gene expression rather than gene presence, differences between individuals from different phenotypic clusters become larger. This shows that even though there is a conservation of gene types at the genome ends, this does not mean that these conserved positions are used in the same manner across the different phenotypic clusters.

##### Different usage of conserved TFBSs

Figure 4B shows the same URRs as in Figure 3B, the only difference being that only TFBSs bound by their corresponding TF (occurs if that TF is expressed) are shown and unbound TFBSs are given the same grey color as absent TFBSs and gaps. Similar to the case for present versus expressed genes, we see that if we compare present versus bound TFBSs, URRs of individuals belonging to different phenotypic clusters become more different. So, again, event hough URRs for a large number of gene types are conserved across the population, they are used differently in the different phenotypic clusters.

##### Mapping genotypic to phenotypic differences

These results illustrate that limited differences in the presence of gene types and TFBSs can be amplified into considerable differences in TFBSs usage and gene expression pattern and hence phenotype. In Figure 4C we use a cartoon to illustrate how the mapping from genome to gene regulatory network to phenotype allows for such a non-linear relation between genotypic and phenotypic differences. We see that very different genomes can give rise to the same phenotype (Figure 4C1 and 4C2), and that small genotypic differences can lead to both small and large phenotypic differences (Figure 4C3 and 4C4). In contrast, the genotype phenotype mapping in the null is linear. As a consequence there is a only a single genotype to code for each phenotype, and large phenotypic differences will require large genotypic differences.

#### Robustness to recombination

Next, the question is how limited genotypic differences, constrained to the middle regions of the genome, prevent a washout of phenotypic differences, resulting in robustness to recombination and increased hybrid fitness. To answer this question we analyzed the details of all matings occurring, focusing on the genotype, network and phenotype formed after recombining the parental genomes into an offspring genome but prior to any further mutations occurring on this genome, and keeping track of nearest niches, phenotypes, and genomic recombination locations of the parents.

**Figure 5:**
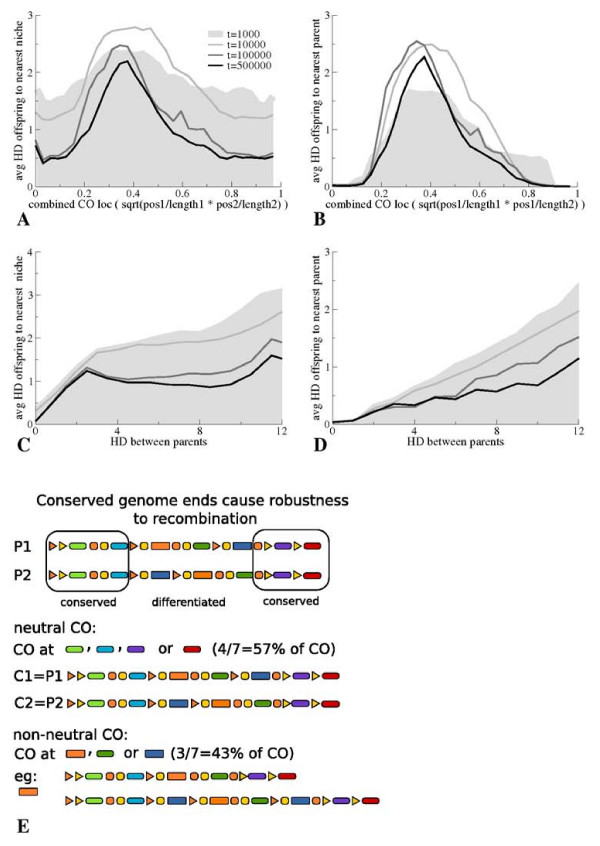
**Evolution of Robustness to Recombination under Canalization**. **A **Evolution of the average phenotypic hamming distance of offspring to the nearest niche as a function of recombination (crossing over, CO) location. We construct a single measure for the CO locations on the two parental genomes: , with *l*_1 _and *l*_2 _locations on the genomes of parent 1 and 2, respectively, and *l *relative CO locations (position of gene at which CO occurs normalised to the number of genes in the genome). If the CO locations on both parental genomes lie at the 'begin' of the genomes (eg. *l*_1 _= 0.1 and *l*_2 _= 0.1) a low value arises, if both locations lie at the 'end' of the genomes (eg. *l*_1 _= 0.9 and *l*_2 _= 0.9) a high value arises. Extreme values thus correspond to recombinations between either two genome 'begins' or two genome 'ends', that is two "corresponding" genome ends. Intermediate values arise if CO locations at opposite ends are used (eg. *l*_1 _= 0.2 and *l*_2 _= 0.8) or if CO locations at the middle regions of the genome are used (eg. *l*_1 _= 0.5 and *l*_2 _= 0.5). **B **Evolution of the average phenotypic HD of offspring to its closest resembling parent as a function of recombination locations on the parental genomes. **C **Evolution of the average phenotypic HD of offspring to the nearest niche as a function of phenotypic HD between the two parents. **D **Evolution of the average phenotypic HD of offspring to its closest resembling parent as a function of phenotypic HD between the two parents. **E **Cartoon showing how conserved genome ends cause robustness to recombination and high hybrid fitness. Two parents adapted to different niches have genomes that are conserved at the ends but differentiated in the middle regions (top). If for recombination a gene is chosen that lies on a genome end, recombination replaces a part of the genome of one parent with a similar part of the genome of the other parent. The offspring genome will thus correspond to the parental genome from which it received the largest, and hence the specific, part (middle). As a consequence, the offspring will be equally well adapted as this parent. If, in contrast, a gene from the middle genome region is chosen, recombination results in the exchange of non-equivalent genome parts between the parental genomes. In this case offspring genomes will contain a mixture of the specific parts of their parental genomes (bottom), causing offspring phenotype to be a non adaptive amalgam of the parental phenotypes. As conserved genome ends take up a considerable part of genome length (here 4 of the 7 genes), a considerable fraction of recombination events will lead to fit hybrid offspring closely resembling one of their parents.

In Figure 5 we display the evolution of offspring fitness and of offspring resemblance to their nearest parent as a function both of the genomic locations used for recombination between the two parental genomes to create the offspring genome (Figures 5A and 5B) and as a function of the phenotypic distance between the two parents (Figure 5C and 5D). The grey shaded area indicates the situation at the begin phase of evolution (t = 1000), whereas the black line shows the situation at the end of the evolutionary simulation (t = 500000).

##### Recombination location and fitness

Figure 5A and 5B show the average HD of offspring to the nearest niche (reflecting fitness) and the average HD of offspring to their nearest parent (reflecting resemblance to one of their parents), respectively, as a function of genomic recombination locations used. We see that over evolutionary time, as genotypic canalization increases and genome ends become more similar (Figure 3), offspring resulting from the recombination between two corresponding genome ends (i.e. on both parental genomes the recombination location is at the 'begin' end or at the 'end' end of the genome) become more and more fit and come to more and more closely resemble one of their two parents. In contrast, offspring resulting from recombinations between non-corresponding genome ends or between middle genome regions become less fit and come to less closely resemble either one of their parents. Put simply, as genome ends become more conserved, recombination events between corresponding genome ends will just swap similar genome regions for one another. Offspring created from such a recombination event will closely resemble on of its two parents (the one from which they receive the largest and hence also the specific part of the genome) and will be equally well adapted as that parent. Such recombination events will thus have a neutral effect on offspring fitness.

##### Hybrids and fitness

Figures 5C and 5D show similar results, but now as a function of the phenotypic distance between the two parents. As to be expected, we see that the hybrid offspring of more dissimilar parents is less fit and less similar to one of its two parents. However, we also see that over evolutionary time, as genotypic canalization arises and genome ends become more similar (Figure 3), it are especially these offspring from more dissimilar parents which fitness and resemblance to one of their parents increases significantly. These results can be easily understood as follows: as over time canalization evolves, those offspring that arise from recombinations between corresponding and hence similar genome ends will be closely resembling one of their parents and be as well adapted as that parent, as we explained above. So, even when very dissimilar parents mate, a significant fraction of all potential recombinations between their genomes will involve the swapping of corresponding and hence similar genome ends. As a consequence, a considerable fraction of these matings will result in fit hybrid offspring, closely resembling one of their parents. The result is an increase in average hybrid fitness and the evolution of robustness to recombination.

To illustrate this mechanism more clearly we show in Figure 5E a cartoon of offspring created from potential recombinations between two differing parental genomes. The two parents are adapted to different niches, which is reflected in their similar genome ends but different middle genome regions (top). If we randomly draw a gene type for a recombination location (see Methods) for a number of times, a large fraction of these recombination events will involve genes lying on the conserved genome ends (middle). As a consequence, a large number of recombinations amount to the swapping of two similar genome ends and hence to the creation of fit offspring genomes closely resembling one of the two parental genomes (middle). Only a subset of potential recombination events lead to offspring genomes that are an unfit amalgam of the two specialized parental genomes (bottom). This results in a high average hybrid fitness and a significant robustness to recombination destroying phenotypic divergence.

#### Polymorphism

Summarizing, our results show how the selection pressure for increased hybrid fitness in the full model with its flexible genetic architecture and non-linear genotype phenotype mapping becomes translated into a selection for genotypic canalization. This genotypic canalization, characterized by conserved genome ends and specific middle regions, subsequently leads to robustness to recombination and high hybrid fitness, thus enabling discrete phenotypic divergence. Both genome structure and network architecture play an essential role in this process. In contrast, in the null model, with its fixed genetic architecture and linear genotype phenotype mapping, no genotypic canalization can occur. Significant phenotypic differences will necessarily require significant genotypic differences, causing recombination to remain equally destructive and hybrid fitness to remain low, thus preventing the accumulation of discrete phenotypic differences. Interestingly, contrary to the common assumption that flexible genetic architectures causing architectural genotypic differences lead to incompatibilities and reproductive isolation, here we observe the complete opposite behavior. The flexible genetic architecture allows for genotypic differences but at the same time constrains their localization and amount, thus increasing hybrid fitness and supporting polymorphic differentiation rather than speciation. In the next section we will investigate the interaction of this mechanism with the evolution of assortative mating, to study whether it also enhances the evolution of sympatric speciation.

### Phenotypic diversity under evolving assortative mating

#### Phenotypic divergence

In models with fixed genetic architectures and linear genotype-phenotype mappings it has been shown that assortative mating and sympatric speciation are necessary for the evolution of distinct phenotypic clusters. Here we have shown that for a flexible genetic architecture and non-linear genotype phenotype mapping distinct phenotypic polymorphisms can evolve under random mating. An interesting question is whether the flexible architecture and non-linear mapping can also make a contribution to the evolution of phenotypic divergence under assortative mating.

**Figure 6:**
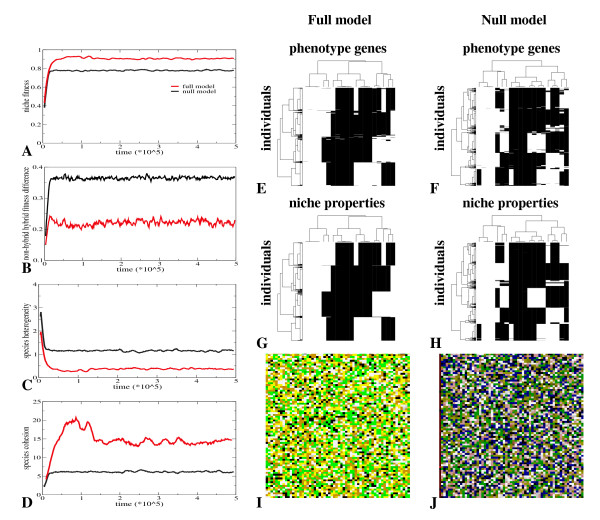
**Evolution of phenotypic diversity under evolving assortative mating**. For an explanation of terms and measures see Methods. **A **Evolution of niche-fitness (equation 2) in the full and null model. **B **Evolution of the difference in niche-fitness between hybrids and non-hybrids in the full and the null model. **C **Evolution of species heterogeneity in the full and the null model. **D **Evolution of species cohesion in the full and the null model. **E **and **F **Clustering of phenotypes present in the population at time 500000 in the full (E) and null model (F). A random 10% sample of individuals are taken from the population. Individuals (rows) are clustered based on phenotype similarity, phenotype genes (columns) are clustered based on across population expression pattern similarity. **G **and **H **Niches to which individuals are best adapted at time 500000 in the full (G) and null (H) model, for the same individuals as in E and F. Row and column clusterings are the same as in E and F. **I **and **J **Snapshots of the distribution of individuals on a 25% part of the grid world at time 500000 in the full (I) and null (J) model.

In Figure 6 we compare the evolution of phenotypic diversity and speciation if assortative mating is allowed to evolve in the full and the null model. We see that in both models niche fitness increases substantially during the first phase of evolution (Figure 6A), but with larger values being reached in the full model. We can also see that in the full model fitness differences between hybrid and non-hybrid individuals are considerably smaller (Figure 6B). In addition, we observe that species heterogeneity decreases (Figure 6C) and species cohesion increases (Figure 6D), again more so in the full model than the null model. In agreement with these findings we see that at time 500000 the population consists of clearly distinct and rather homogeneous phenotypic clusters, 4 in the case of the full model and 5 in the case of the null model (Figures 6E and 6F), and that individuals from the same cluster have specialized on the same niche (Figures 6G and 6H). In Figures 6I and 6J we see that the different species occur well mixed over space. If we compare these results with the results from Figure 2 we conclude that in the presence of assortative mating discrete phenotypic diversity now can evolve in the null model, as expected. Quite strikingly, the level of niche fitness, species cohesion and phenotypic clustering evolved in the null model under assortative mating is very similar to that evolved in the full model under fully random mating. If we compare the results from the full model and null model for assortative mating we see that fitness levels attained in the full model are higher, with smaller and decreasing fitness differences between hybrids and non-hybrids, with lower species heterogeneity, higher species cohesion and more distinct, more homogeneous phenotypic clusters.

The increased levels of hybrid fitness and species cohesion in the full model can be explained by the occurrence of a similar genotypic canalization process as described before under random mating (see Additional file 1, Figures S3 and S4).

#### Effective Assortativeness

**Figure 7:**
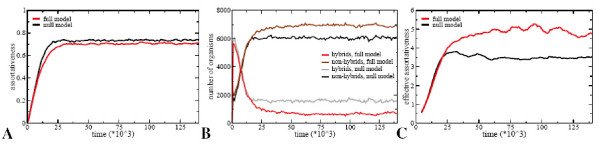
**Evolution of assortative mating**. Results are averages over 5 simulations, and compare assortativeness evolving in the full and null model. **A **Evolution of the average assortativeness level in the population. **B **Evolution of the number of hybrid and non-hybrid organisms in the population. **C **Evolution of *effective assortativeness*. Effective assortativeness is defined as the fraction of matings that occur between individuals belonging to the same species.

How do these differences affect the assortativeness evolving in the full and the null model? To be able to study this we need to make a distinction between *assortativeness*, which reflects the preference strength of individuals for like or dislike mates (independent of availability), and *effective assortativeness*, which reflects the fraction of matings in which individuals mate with individuals belonging to the same species (assuming positive assortativeness: a preference for like individuals, which are individuals belonging to the same species). Clearly, the number of species present in the population influences the effectiveness of assortativeness as it affects the chances for an individual to meet like or dislike individuals in its neighborhood. Therefore, to perform a fair comparison, we ensured that compared simulations from the null and full model contained the same number of species. Figure 7A shows the evolution of average assortativeness in both models. We see that in the full model slightly lower levels of assortativeness evolve. However, Figure 7B shows the evolution of numbers of hybrid and non-hybrid organisms in the population. Thus, despite the slightly lower level of assortativeness there are less hybrids in the full model. In Figure 7C we display the evolution of effective choosiness. We see, consistent with the lower number of hybrids, that effective choosiness evolves to a significantly higher level in the full model. So, with similar preference strength for like individuals and with similar chances of meeting like individuals, in the full model individuals are better able to preferentially mate within their species.

#### Linking canalization and effectivity of assortativeness

Above we have shown that in the full model, due to the genotypic canalization process, species cohesion is larger. In addition we found that the effectiveness of being assortative is larger in the full model. How are these two findings linked? In our model, mate choice is based solely on phenotypic differences between the individual and it's potential mates (see Methods, equation 3). There is no information available on whether or not the potential mate belongs to the same species. This implies that the effectiveness of assortativeness, how often an individual is able to mate with individuals belonging to the same species, strongly depends on how well phenotypic differences reflect species membership. The latter depends on species cohesion, if species cohesion is strong, i.e. individuals of the same species are very similar and individuals of different species are very dissimilar, phenotypic differences are a high quality measure for species membership and the effectiveness of being choosy will be high. So, in the full model genotypic canalization leads to increased species cohesion (Figures 2D and 6D) and hence to an increased effectiveness of being assortative.

#### Speciation

Summarizing, we obtain the interesting but counterintuitive result that by increasing hybrid fitness, thus increasing species cohesion and effectiveness of being assortative, the reproductive isolation between species is enhanced.

### Generality of Results

We explained in detail the mechanism that, in the full model, enables phenotypic differentiation under random mating and enhances it under assortative mating, focusing on a few typical simulations in which default model parameter settings were used. We showed that the flexible genetic architecture and non-linear genotype phenotype map of our full model allowed for a genotypic canalization process that restricts the amount of genotypic differences underlying phenotypic differentiation. As a consequence, robustness to recombination evolves, hybrid fitness increases and phenotypic divergence arises despite random mating. Under assortative mating this same process enhances phenotypic divergence and increases the effectiveness of being assortative.

To determine the generality of these results we performed a large number of additional simulations, varying initial conditions, parameter settings and even major building blocks of our model. In all cases we observed the evolution of distinct phenotypic clusters, restricted genotypic variation, increasing hybrid fitness and increasing robustness to recombination under random mating. In case of assortative mating, we observed in all cases enhancement of phenotypic divergence and of the effectiveness of being choosy. We are therefore confident that our results do not depend critically on the specific details of our model. Details of these additional simulations can be found in Additional file 1.

## Discussion

### Results

In this paper we investigated the role of a evolving genome organization and gene regulatory network architecture and a non-linear genotype phenotype mapping on the evolution of phenotypic diversity in sexually reproducing populations. For this purpose we constructed a model in which individuals contain a genome with transcription factor genes, phenotype genes and transcription factor binding sites. The genome determines a gene regulatory network architecture, which together with an initial birth state determines the gene expression pattern and hence phenotype of the organisms. Mutations occur on individual genes, transcription factor binding sites and stretches of genome. For comparison purposes, we also constructed a null model in which organisms contain a simple genome with only phenotype genes and mutations occurring on the state of the genes.

We found that in the full model different morphotypes well adapted to different niches evolved despite fully random mating. We showed that the selection pressure for increasing hybrid fitness translates, in the presence of the flexible genetic architecture and non-linear genotype phenotype map of the full model, into a selection for genotypic canalization. We demonstrated that this genotypic canalization leads to a structuring of the genome such that differences are constrained to the middle regions and genome ends are conserved. We next showed how the non-linear genotype phenotype mapping of the full model allows for full blown phenotypic divergence despite this constrained genotypic differentiation. Finally, we demonstrated that, by having conserved genome ends, a significant fraction of potential recombinations will swap equivalent genome ends. We showed that, as a consequence, a large fraction of hybrids offspring closely resembles one of the two parents and is equally well adapted as that parent, rather than being an unfit amalgam of the properties of the two parents. This shows how by constraining genotypic differentiation an increased robustness to recombination and increased hybrid fitness can arise.

Note that the conserving of genome ends and differentiation of middle genome regions may be the result of specific model choices we made. One can imagine that if a varying number of recombination events between parental genomes are allowed, or if genomes consist of multiple chromosomes, different regionalizations of conserved and varying regions may arise. The main point we want to make here is that by having a flexible genetic architecture and a non-linear mapping, genotypic differences can be constrained causing hybrids to become fitter, and not so much where exactly conserved and varying regions lie on the genome.

In agreement with results from other modelling studies, in the null model the population was unable to diversify such that different types of organisms well adapted to different niches arose under random mating. We found that when assortative mating was allowed to evolve, significant levels of assortativeness and sympatric speciation arise in both models. Even though the assortativeness level evolved in both models is similar, effective assortativeness levels are higher in the full model. We show that under assortativeness, despite the much lower rate of hybrid formation, genetic canalization still evolves. This canalization process increases species cohesion, thus resulting in more effective choosiness.

### Predictions and future work

Based on our study results we can make a number of predictions. The first prediction is that the evolution of phenotypic differentiation can precede the evolution of any significant level of assortativeness (see Figure S9B, Additional file 1). This is in strong contrast with results from previous modelling studies [5-9], which predicted that differentiation and assortativeness by necessity arise simultaneously.

A second prediction of our model study is that hybrids of species arisen by sympatric speciation should be rather fit. In agreement with this prediction, a recent study on the fitness of hybrids of cichlids species pairs in the Lake Victoria flock reported no significant fitness differences between species and their hybrids [52]. Note that it is often thought that the evolution of incompatibilities that cause hybrid inviability or sterility lead to reproductive isolation and species formation. However, Darwin already realized that although such properties may be advantageous at the species level, for an individual there is no selective advantage for properties that reduce the viability or fecundicity of part of its offspring [53]. Therefore, we propose that hybrid inviability and sterility usually arise as the by products of allopatric or parapatric speciation processes, or arise in later stages of sympatric speciation. In other words, hybrid inviability and sterility are more likely to be a result of, rather than a cause for speciation. For the early stages of sympatric speciation, in which assortativeness is incomplete and reduced hybrid viability or fecundicity would impose large fitness costs, they should not be expected.

A third prediction is that we would expect more synteny of genes between sympatrically than parapatrically arisen species. In addition, we would expect that species or morph specific genes are more closely linked on the genome of sympatric than parapatrical species. The existence of supergenes, a cluster of closely linked genes, in certain genetic polymorphisms [12,13,37] supports this prediction.

Finally, we hypothesis that the canalization process occurring in the full model increases the likelihood of the evolution of assortative mating and sympatric speciation in two ways, making sympatric speciation more likely than it is often considered to be. First, it allows for discrete phenotypic variation despite random mating, increasing the diversity of phenotypes to choose from at the beginning of the evolution of assortativeness. Second, it enhances the effectiveness of any amount of assortativeness arising. In a future study we plan to test this hypothesis by studying the evolution of assortative mating under conditions where it is far less easy for assortativeness to evolve (see eg. [1,5,7,14-16]) to see whether indeed assortativeness can evolve in the full model under conditions where it can not evolve in the null model.

### Relation to other research

Our finding that under sexual reproduction robustness to recombination evolves agrees with findings from previous modelling studies, also using a non-linear genotype phenotype mapping, in which sexual reproduction was shown to enhance the evolution of robustness to recombination [54-56]. In our study we have shown that the mechanism behind this increased robustness to recombination is a canalization of genome and network organization, limiting differences to certain locations.

Similar results have been reported in population genetics studies in which the epistasis between loci was allowed to evolve. In these studies it was shown that discrete polymorphism could evolve despite random mating by restricting the amount of genotypic polymorphism to a limited number of genomic loci [1,10]. An important difference between these studies and our study is that rather than allowing an abstract epistasis parameter to evolve, we show mechanistically how, via the interplay between the non-linear genotype phenotype mapping and the flexible genome and gene regulatory network architecture, variation becomes limited to a number of loci predominantly located in the middle of the genome. Furthermore, we thus demonstrated the importance of both a non-linear genotype phenotype mapping (dominance or epistasis in the population genetics models) and of genome structure, the latter of which is absent in the population genetics models.

Intriguingly, in a modelling study by Kaneko, fundamentally different results were found [57,58]. They reported a progressive decrease of hybrid fitness during sympatric speciation, in contrast to the increase in hybrid fitness observed in our model. In their model, speciation critically depends on the initial presence of phenotypic plasticity (a one genotype to many phenotype mapping) and an interaction based instability mechanism. Together, these lead to the situation that in an initially genetically homogeneous setting, phenotypic homogeneity is unstable and phenotypic differentiation occurs. The thus arising phenotypic differences lead to decreasing hybrid fitness, causing the phenotypic differences to become genetically assimilated, further enhancing the process. Similar to our study, they show the ready evolution of assortative mating, which in this case is even stronger selected for due to the larger unfitness of hybrids in this model. The fact that this model allows for genotypic plasticity, which is not the case in our model, may play in important role in the contrasting effects for hybrid fitness observed in their and our model. However, other differences between the two models may be equally important.

Finally, our results may help shed light on the causes behind the observed non-random gene order of eukaryotic genomes [59-65]. Several mechanisms have been suggested to underly the observed ordering. One possibility could be that the non-random order is a mere effect of the mutational dynamics of the evolutionary process [61,63]. Other mechanism that have been suggested are co-expression [59,63,64], dosage balance [62] and the link between genome modularity and mutational and recombinatorial robustness [56,60]. In addition, a link has been demonstrated between genome organization and evolvability [66,67]. Based on our results, we propose a new mechanism behind non-random genome organization. Hybridization between different morphs of a single species or the continued residual hybridization between closely related sympatric species can cause selection for a non-random genome ordering that increases robustness against such hybridizations.

## Conclusion

In models where a fixed genetic architecture and a linear genotype phenotype mapping is assumed, the flexibility of the evolutionary process is severely constrained. In such models the buildup of discrete phenotypic differences in sexually reproducing populations can only occur if mating is assortative. In contrast, in models with a flexible genetic architecture and a non-linear genotype-phenotype mapping, such as the present model, the evolutionary process is much more flexible. Here we demonstrate that evolution goes to part of the genotype-phenotype landscape were only limited genotypic differences are needed to generate phenotypic differences, a process we call genotypic canalization. This canalization leads to robustness to recombination and increasing hybrid fitness, allowing for discrete phenotypic differentiation despite random mating. In addition, under assortative mating effectiveness of assortativeness is enhanced. So, if a flexible genetic architecture and non-linear mapping are assumed, not the phenotypic differentiation, but the genotypic differences underlying this differentiation are constrained under sexual reproduction.

## List of abbreviations used

HD: hamming distance; TF: transcription factor; TFBS: transcription factor binding site; URR: upstream regulatory region.

## Authors' contributions

KT conceived and constructed the model and performed the model simulations. Both authors contributed to the analysis of the results and the drafting of the manuscript. Both authors read and approved the final manuscript.

## Supplementary Material

Additional file 1 **Supplemental material**. In this file additional results can be found. Click here for file
