# Supplementary material for: The role of genome and gene regulatory network canalization in the evolution of multi-trait polymorphisms and sympatric speciation
Source: BMC Evol Biol. 2009 Jul 9;9:159. doi: 10.1186/1471-2148-9-159 (PMC3224660; doi:10.1186/1471-2148-9-159)
Supplement: Additional file 1 — Supplemental material. In this file additional results can be found. [file 1471-2148-9-159-S1.pdf]

Supplementary material accompanying "The role of genome and gene regulatory network canalization in the evolution of multi-trait polymorphisms and sympatric speciation"

Ten Tusscher K. H. W. J. and Hogeweg P.

For quantitative measures of the genome and network architecture canalization we introduce a number of additional terms used in this supplementary material:

Gene type similarity: the frequency of the most common gene at a particular position in the population level genome alignment. Measures the population's homogeneity with respect to the gene type present at that genome location.
A histogram of gene type similarities of all aligned genome positions represents overall **genome order similarity**: how many positions are how homogeneous in the population.
Gene type similarity, genes present: all genes present at an aligned genome location are counted to determine the most common gene type.
Gene type similarity, genes expressed: only genes that are present and expressed at an aligned genome location are counted to determine the most common gene type. This results in measure of gene type and genome order similarity that is more stringent, similarity is only high if a large number of individuals have and express the same gene types at the same genome locations.

Binding site similarity: the frequency of the most frequent binding site in the population at a particular position in the upstream region of a particular gene.
Upstream region similarity: average of the binding site similarities in the upstream region of a gene. A maximum of 3 binding sites are counted per gene. If less binding sites are present, remaining ones are considered empty, if more are present they are ignored.
A histogram of upstream region similarities represents overall **network architecture similarity** how many genes have how similar regulatory interactions with other genes in the population.
Binding site similarity, binding sites present: all binding sites present in a particular upstream region location (position 1, 2 or 3) are counted to determine the most common type.
Binding site similarity, binding sites occupied: only binding sites present and occupied by a TF are counted to determine the most common type. This results in measure of binding site, upstream region and network architecture similarity that is more stringent, similarity is only high if a large number of individuals have and use the same binding sites for the same genes.

Genotypic canalization under random mating

Figure S1 shows the genome and network differentiation underlying phenotypic differentiation in the full model under asexual reproduction. We see that genome order (Fig. S1A)

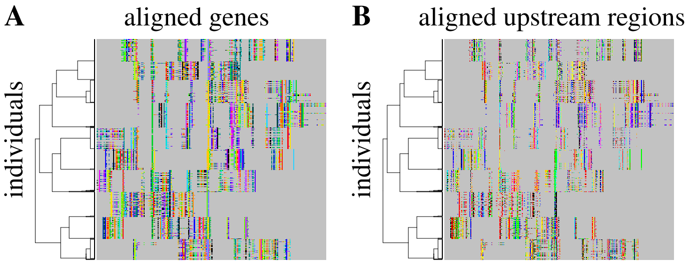

Figure 1: **A** Aligned genome order under asexual reproduction. Alignment is performed using Clustalw (see Methods) Grey: gap in the alignment; colors: different gene types. Organisms are a random 10% sample of the population at time 500000 and are clustered based on phenotype. **B** TFBS present in the upstream regulatory regions of genes. We show a maximum of 3 binding sites per gene. Genes are ordered based on their position in the genome alignments as shown in A. Grey: absent TFBS (gene has less than 3 TFBS) and alignment gaps; colors indicate type and weight of TFBS. Organisms shown are the same as in A and are again clustered based on phenotype.

and upstream regulatory regions (Fig. S1B) are similar within morphs (which correspond to clonal species under asexual reproduction). However, in strong contrast to the situation under sexual reproduction (Figure 3B and 3C), genome order and network wiring between different morphs are completely different, rather than these differences being constrained to the middle region of the genome.

In Figures S2A-F we quantify the canalization occurring under sexual reproduction and compare it to the situation under asexual reproduction. In Figure S2A and S2D we use gene type similarity histograms to study genome order similarity. We see that for random sexual reproduction (Figure S2A) a considerable frequency of genome positions ($\sim 25\%$) have a high gene type similarity score (0.8 or higher), indicating that a significant part of the genome is highly canalized across the population. In contrast, for asexual reproduction (Figure S2D) most genome locations have a similarity score of 0.2 or less. However, if we consider gene type similarity histograms for genes present and being expressed the distributions are much more similar, and in both cases hardly any genome positions with similarity scores above 0.2 are present. Under sexual reproduction, canalization thus occurs on the gene types present, but these similar gene types are subsequently being differently expressed by different individuals.

To study regulatory network similarity, we use upstream region similarity histograms (Figures S2B and S2E). Under random sexual reproduction (Figure S2B) there is a high frequency of gene types with intermediate to high similarity for upstream regulatory regions, indicating considerable across population canalization for a large part of the network. In contrast, for asexual reproduction (Figure 2E) most gene types have upstream region similarity scores of 0.2 or less. However, if we consider upstream region similarity histograms for TFBS present and occupied, these distributions become less dissimilar. We see that under random sexual reproduction the frequency of genes with very dissimilar upstream regions increases and the frequency of genes with very similar upstream regions drops strongly. So again, similarity is mainly in the presence and composition of upstream regulatory regions, not so much in their usage.

Figures S2C and S2F show the correlation between gene type and upstream region similar-

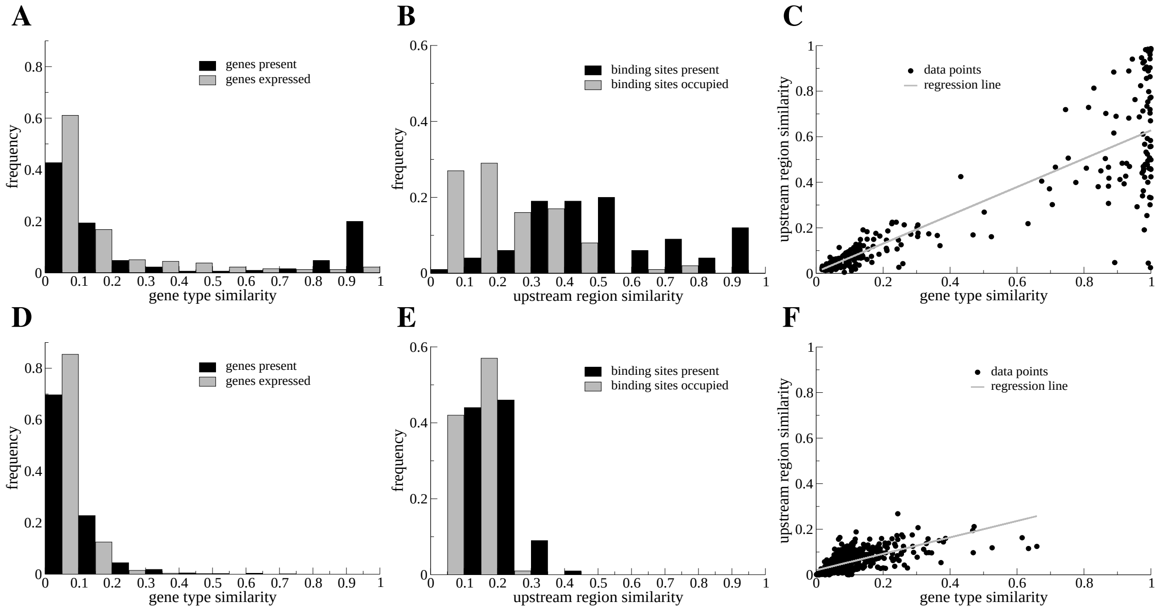

Figure 2: Quantitative genome order and network similarity in the full model for random sexual and asexual reproduction. Results are from 5 simulations for random sexual reproduction and 5 simulations for asexual reproduction. For an explanation of terms see Methods. **A** and **D** Genome order similarity for sexual (A) and asexual (D) reproduction. **B** and **E** Network architecture similarity for sexual (B) and asexual (E) reproduction. **C** and **F** correlation between gene type similarity and upstream region similarity for random sexual (C) and asexual reproduction (F). Note that upstream region similarity now is computed over genes with the same position in the alignment, rather than over genes of the same type.

ity at a genome location. For sexual reproduction (Figure S2C) there is a strong correlation between gene type and upstream region similarity, consistent with the results in Figure 3C, which showed that at positions were gene type was conserved the upstream regulatory regions also tended to be conserved. Most genome positions are either very dissimilar or very similar for both gene type and upstream region, with few intermediate positions. For asexual reproduction, all genome positions have low similarity scores for both gene type similarity and upstream region similarity (Figure S2F), in agreement with their overall rather than constrained genotypic divergence (Figure S1).

Genotypic canalization under assortative mating

Figure S3 shows the evolution of phenotypic differences, genome order differences and network wiring differences for the full model under assortative mating. We see that the amount of genome and network differences correspond more closely to those evolving under random sexual reproduction than under asexual reproduction, suggesting a similar amount of genotypic canalization under assortative as under random mating..

These results are further confirmed in Figure S4. Figure S4 shows the gene type and upstream region similarity and their correlation in the full model under evolving assortative mating. We see a similar pattern as for the case of random mating. A substantial number of gene positions (35%) has high gene type similarity (0.8 or higher) (Figure S4A), and a substantial number of gene types (80%) have intermediate to high upstream region similarity (0.3 and higher) (Figure S3B). This similarity decreases substantially when we consider

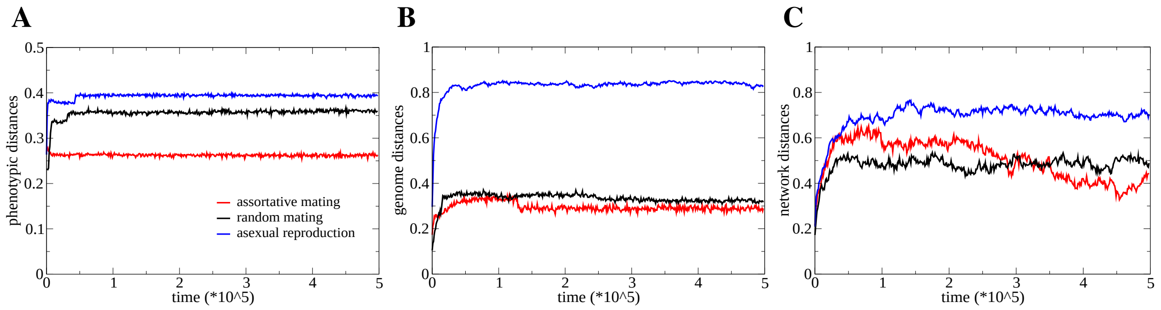

Figure 3: Evolution of differentiation under evolving assortative mating. Evolution of average, population level phenotypic distances (**A**), genome distances (**B**) and network distances (**C**) under evolving assortative mating. For comparison purposes we also show the distances evolving under random mating and under asexual reproduction, which are the same as those shown in Figure 3A of the main article. For an explanation of the measures see the Methods section of the main article.

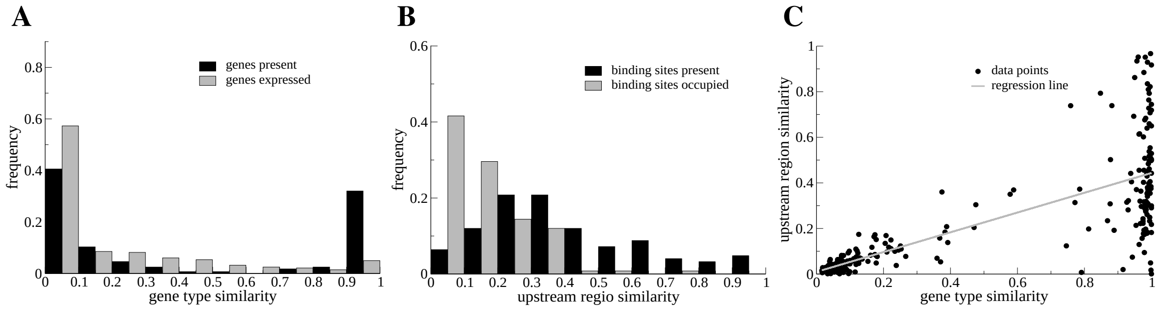

Figure 4: Genome order and upstream region similarity in the full model under assortative mating. Results are from a total of 5 simulations, at time 500000. **A** histogram of gene type similarity. **B** histogram of upstream region similarity. **C** correlation between gene type similarity and upstream region similarity.

expressed rather than present genes (Figure S4A), and occupied rather than present transcription factor binding sites (Figure S4B). There is a strong positive correlation between gene type and upstream region similarity, with most genome locations having either high or low similarity levels for both properties (Figure S3C).

So clearly, despite the evolving assortativeness and hence decreasing levels of hybrid offspring formation, a comparable amount of genotypic canalization occurs. However, in our model evolved levels of assortativeness remained well below 1 for the default parameter settings (see Figure 7). A question thus is whether genotypic canalization still arises if higher levels of assortativeness evolve, leading to much lower numbers of hybrid offspring and hence considerably less selection for increased hybrid fitness and genotypic canalization.

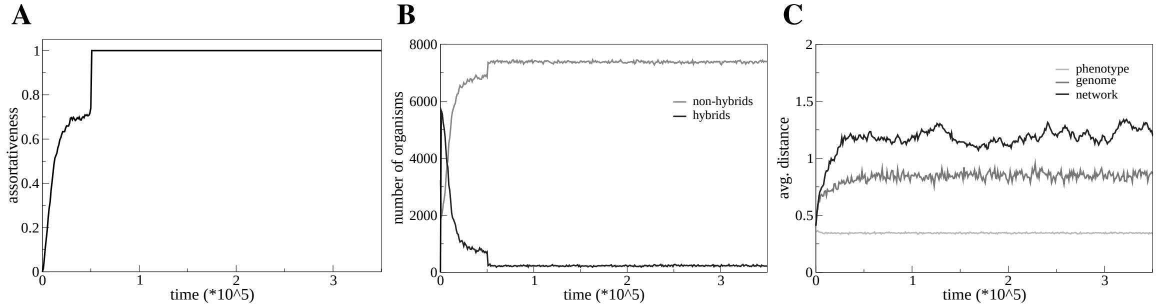

Figure 5: The first 50000 time steps normal evolution of assortativeness occurs, after that assortativeness of all individuals in the population is fixed to the maximum value of 1. **A** Average population level assortativeness over time. **B** Number of hybrid and non-hybrid organisms in the population over time. **C** Population level phenotype, genome and network differences over time.

Figure S5 shows the results of a simulation in which after an initial period of normal evolution of assortativeness, the assortativeness of all individuals is fixed to the maximum level of 1 at time 50000 (Figure S5A). We see that this results in an decrease of hybrid and an increase of non-hybrid individuals (Figure S5B), with hybrids now only accounting for around 3% of the total population. We see that similar levels of genome and network differences evolve and persist to drive phenotypic differentiation as is the case if assortativeness is allowed to evolve normally during the entire simulation (Figure 11).

These results demonstrate that genotypic canalization evolves even under high assortativeness levels and hence low numbers of hybrid offspring.

Robustness of results

To determine the generality of the results reported in the main article we performed three series of additional simulations. In the first series initial conditions were varied, in the second series parameters were varied, and in the third and final series substantial building blocks of the model were varied. In all cases we observed discrete phenotypic and genotypic divergence, increased robustness to recombination and increased fitness of hybrids, implying that the results we presented in the main article are robust and general.

Dependence on initial conditions

To investigate the dependence of our results on initial conditions we varied the initial genome the population starts with (3 simulations), the birth-state networks start their dynamics with

(4 simulations), the 20 random niches organisms can adapt to (4 simulations), and the seed used for the random number generator to draw mutation events (4 simulations).

For these simulations we measured the number of phenotypic clusters that evolved, average niche fitness at the end of the evolutionary simulation, and average difference in this niche fitness between hybrid and non-hybrid organisms (parents adapted to different respectively the same niche), and compared this to the default simulations discussed in the main article. We choose these parameters as they provide some general measure of whether discrete differentiation is occurring (nr of clusters), how well adapted organisms manage to be (fitness), and whether indeed hybrids are not very less fit than non-hybrids, despite random mating. Results of these simulations are summarized in Table S1.

simulation	nr of pheno clusters	avg fitness	avg hybrid non-hybrid fitness diff
default	8	0.69	0.08
genome 1	13	0.72	0.09
genome 2	11	0.68	0.11
genome 3	10	0.69	0.08
birth-state 1	8	0.62	0.10
birth-state 2	6	0.74	0.02
birth-state 3	7	0.66	0.08
birth-state 4	9	0.71	0.05
niches 1	11	0.75	0.05
niches 2	6	0.82	0.01
niches 3	8	0.74	0.02
niches 4	8	0.66	0.09
seed 1	8	0.68	0.06
seed 2	8	0.74	0.05
seed 3	9	0.71	0.06
seed 4	8	0.78	0.03

Table 1: Dependence of model outcomes on initial conditions

We see no significant differences in simulation outcome for the different initial conditions tested, and conclude that there is no dependence of our results on the initial conditions.

Dependence on parameter settings

We investigated the dependence of our results on the parameter settings used by varying the number of niches (14 or 7 instead of 20), the size of the neighborhood radius in which organisms interact (4 and 1 instead of 2), the strength of the competition parameter (β 100 or 25 instead of 250), the rate of mutations (10 times higher or 10 times lower), and the maximum number of update steps before the phenotype is determined (30 or 60 instead of 10 iterations). Results of these simulations are shown in Table S2.

Apart from some quantitative differences, no qualitative changes in model behavior are observed: we get differentiation into discrete phenotypic clusters and high levels of hybrid fitness. We thus conclude that our model outcomes do not critically depend on the parameter settings used.

Dependence on major model building blocks

Next we investigated the dependence of our results on major assumptions and building blocks in our model.

simulation	nr of pheno clusters	avg fitness	avg hybrid non-hybrid fitness diff
default	8	0.69	0.08
14 niches	4	0.69	0.02
7 niches	5	0.75	0.03
radius 4	17	0.62	0.09
radius 1	6	0.75	0.04
comp. strength 100	6	0.63	0.10
comp. strength 25	4	0.77	0.05
mut. rate $\times 10$	11	0.51	0.11
mut. rate $\times 0.1$	7	0.75	0.04
30 iterations	8	0.77	0.03
60 iterations	7	0.75	0.03

Table 2: Dependence of model outcomes on parameter settings

Fitness function
The shape of the fitness and competition function is known to potentially have important consequences for the outcome of the evolutionary process. To investigate whether this is the case for our model we varied these functions. First, we made fitness only dependent on the nearest niche, rather than all niches nearer than a certain threshold hamming distance, the shape of the functions was kept the same. Next, we varied the values of the coefficients α and γ from 0.8 and 1.0 to 0.4 and 0.6, respectively. in the fitness and competition functions. Finally, we replaced the exponential fitness and competition function by Gaussian functions ($e^{-HD*HD/2*s*s}$, with s 1.4 for niche fitness and 1.2 for competition) or by power functions $((1 - HD/nrgenes)^n$, with n 10 for niche fitness and 12 for competition).

Again, apart from some quantitative variation in the number of clusters formed (respectively 7, 12, 9 and 11) and the average fitness levels (respectively 0.75, 0.76, 0.63 and 0.72), qualitative model behavior remained unchanged.

Global interactions

A second major part of our model are the locality of interactions between individuals. There-

A

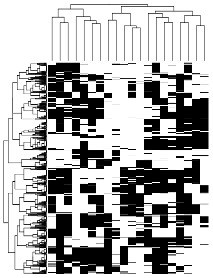

B

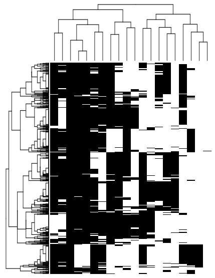

Figure 6: Phenotypic clusters evolved at the end of simulations in which interactions between organisms were global rather than local. **A** Default parameter settings. **B** Default parameter settings, except for β: 50 instead of 250.

fore, we investigated the impact of global rather than local interactions on our results. Figure S6 shows snapshots of the phenotypic diversity evolved at the end of 2 simulations in which competition and reproduction were global. In Figure S6A results are shown when, other

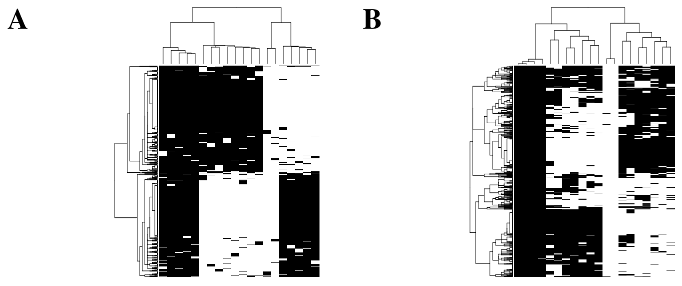

Figure 7: Phenotypic diversity for the model if the number of niches is 2. **A** Full model **B** Null model.

than the global nature of the interactions, default parameter settings were used. We see the evolution of discrete phenotypic clusters. However, the number of clusters is considerably larger, and their homogeneity is considerably less than in the case of local interactions. This can be understood from the fact that there is a stronger selection pressure for differentiation when competing with the entire population than when competing only with a few local neighbors. This higher selection pressure for differentiation leads to more phenotypic clusters, the higher number of different clusters in its turn leads to a larger amount of hybrid matings and hence less homogeneity of the different clusters. To compensate for the higher pressure for differentiation, we studied in Figure S6B the phenotypic diversity arising under global interactions if β, the competition strength, is lowered from 250 to 50. In this case, we see a comparable number and homogeneity of phenotypic clusters as for the case of local interactions, and evolution of comparable average fitness values (0.59).

Birth-state

In the default model we use a single constant birth state as initial gene expression pattern for all individuals in the population, randomly generating this birth state at the beginning of the simulation. To investigate the importance of this modelling choice for our results we performed simulations in which individuals inherit the expression state of genes from their parents with a low error rate and use this as an initial gene expression pattern.

We find a similar number of discrete phenotypic clusters (9) as for the default situation, but with a relatively low average fitness value (0.59). The latter is most likely due to the less predictable birth-state of individuals due to inheritance with errors rather than a constant birth-state.

Two niche case

A fourth important part of our model is the presence of a large number of alternative optimal niches in the organisms environment (typically 20) on which they can differentiate. In classical model studies typically a small number of niches has been studied [5-9]. In modeling studies by Doebeli and co-workers [5,7], the single niche situation has been used as a baseline for studying sympatric speciation, whereas in modeling studies by Kondrashov and Gavrilets [6,8,9], the two niche case has been used as the baseline situation. Our model can be seen as an extension of these two niche models, simply having more than two optimal niches. In contrast, our model is substantially different from the one niche case, in which differentiation requires deviation from the optimal niche and discrete differentiation is known to occur only for particular parameter settings [5,7]. Therefore, the two niche situation would be a natural baseline study for our model. Note that in this case there is no freedom as to which subset of the present niches will be used by the population to differen-

tiate on to avoid too much competition, instead the 2 niches that are present have to be used in order to differentiate.

Figure S7A and B shows the phenotypic diversity evolved in this setting for the full model and null model, respectively. In the full model we can clearly see the presence of 2 distinct phenotypic clusters, in agreement with the presence of 2 niches. So, as for the 20 niche situation, the flexible genetic architecture and genotype phenotype mapping are used to reduce the deleteriousness of recombination and produce hybrid offspring that closely resemble one of their two parents rather than being an unfit amalgam of these two parents. In contrast, in the null model we see a larger number of phenotype clusters than there are niches. The additional phenotypes are the result of recombination persistently mixing the 2 niche phenotypes into non-fit intermediate hybrid phenotypes.

Note that in the models of Kondrashov and Gavrilets [6,8,9], in the absence of assortativeness, no clear phenotypic clusters can evolve. In contrast, we see the formation of several quite clear phenotypic clusters in our null model under random mating, even though some of them do not correspond to a niche phenotype, but to hybrids formed between niche phenotypes. In the main article we showed that if there are 20 niches in the environment, in the null model under random mating no discrete phenotypic clusters could evolve. Apparently, if there are only 2 niches in the environment, the continuous formation of non-fit hybrids is not enough to prevent the formation of discrete phenotypic clusters. Our null model lacks the flexible genetic architecture and gene regulatory network of the full model, but does have its genes linked on a genome, in contrast to the models of Kondrashov and Gavrilets were genes are unlinked. This indicates that, apart from the large role played by the flexible genetic architecture and non-linear genotype phenotype mapping of the full model, even having a static genome architecture can already contribute to the evolution of phenotypic divergence under random mating, although in a lesser manner.

Separating genome order and network wiring

A fifth important part of our model is the mapping from genome to regulatory network to

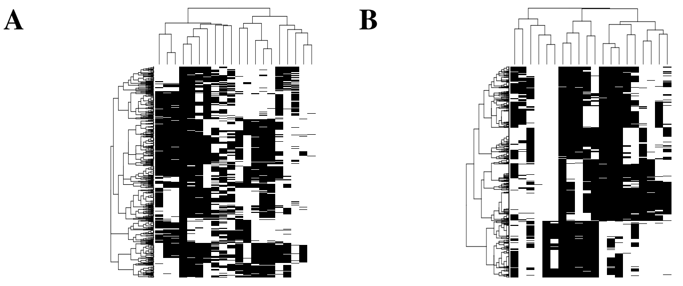

Figure 8: Phenotypic clusters evolved at the end of simulations **A** Only genome order is allowed to evolve, no network wiring incorporated.**B** Genome order and gene copy numbers are fixed, only network wiring is allowed to evolve.

gene expression pattern, where both the genome order and the regulatory network wiring could evolve. To investigate the influence and importance of these two major components we simplified our model in two alternative ways. First, we performed simulations in which organisms have a genome which order and redundancy can evolve, and with genes which expression state can evolve, such that the genome directly codes for the phenotype rather than via a regulatory network determining gene expression. Second, we performed simulations

in which genome order and redundancy are fixed, but there is a network wiring present that functions as an intermediate, mapping the genome to the phenotype, and which can evolve. We see that if only genome order can evolve (Figure S8A), still discrete phenotypic clusters can evolve, but the homogeneity of the clusters is less than in the full model. Similarly, if only network wiring can evolve (Figure S8B) we also observe the evolution of discrete phenotypic clusters that are slightly less homogeneous than in the full model. A simple explanation for the observed differences in phenotype cluster quality is that in case only genome order or only network wiring is allowed to evolve there is less non-linearity and redundancy in the genotype phenotype mapping. As a consequence there will be less opportunity for the evolutionary process to go to parts of the genotype phenotype landscape that reduce the impact of recombination.

However, in both cases we observed discrete phenotypic and genotypic divergence, increased robustness to recombination and increased fitness of hybrids. This suggests that a single non-linear, redundant part of the genotype-phenotype mapping is sufficient for genotypic canalization and phenotypic diversification despite random mating to occur. Furthermore, it shows very clearly that it is not just the flexible gene regulatory network architecture but also the flexible genome structure that allow for discrete differentiation under random mating in our model.

Assortativeness

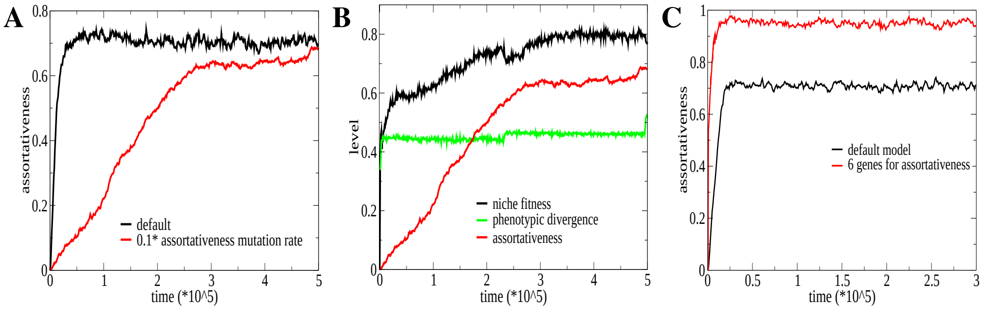

Figure 9: Evolution of assortativeness. **A** Comparison of assortativeness evolving under default parameter settings and if the mutation rate for evolving assortativeness is lowered by a factor 10. **B** Niche fitness, phenotypic divergence and assortativeness evolving if the mutation rate for assortativeness is lowered by a factor of 10. **C** Comparison of assortativeness evolving in the default model and if assortativeness is coded for by the expression of an additional 6 assortativeness genes.

A final important building block of our model of which we investigated the consequences is the precise implementation of assortativeness and it's evolution. The mutation rate used in the main article is quite high, mainly to reduce the computational time needed to complete simulations. We investigated the importance of this by using a 10 times lower mutation rate. Figure S9A shows evolution of assortativeness in the default model and if mutation rates for assortativeness are lowered 10 fold.

As expected, assortativeness evolves considerably more slowly, but otherwise similar results are obtained. More interestingly, in Figure S9B we show the evolution of niche fitness, phenotypic diversity and assortativeness under the setting where assortativeness mutation rates are 10 times as low. We see that niche fitness and phenotypic diversity rapidly increase during the first phase of evolution, when assortativeness levels are still very low. On a slower

timescale, during the increase of assortativeness, phenotypic diversity, but especially niche fitness show smaller and slower subsequent increases. This result clearly shows that the major part of phenotypic divergence and adaptation can precede any significant assortativeness in our model. This is in agreement with our finding that discrete phenotypic differentiation can also evolve in the absence of assortativeness. However, it is in strong contrast with findings from previous modelling studies in which assortativeness is necessary for and occurs thus simultaneously with phenotypic divergence [5-9]. Note that if there are fitness costs for being choosy, if there is noise in observing potential mates, or if mate choice is based on an additional character, not the ecological character under disruptive selection [1,5,7,14,15,16], and assortativeness thus becomes harder to evolve, a situation in which phenotypic divergence precedes the evolution of assortativeness may become even more likely.

There is another interesting point to note here. If we evolve assortativeness with a 10 times slower rate in the null model (data not shown), assortativeness of course evolves slower than for default parameter settings in the null model, but not as slow as we observed for the 10 times lower mutation rates in the full model. So, not only do phenotypic divergence and well adaptedness to the different niches evolve before any significant assortativeness evolves, they actually slow down the evolution of assortativeness. This can easily understood from the fact that in the full model there is less selection pressure for assortativeness to evolve as phenotypic divergence to adapt to the different niches can already occur in the absence of assortativeness.

In the default model we used a separate parameter to code for the level of choosiness of an individual, which is not quite elegant in combination with the multiple genes, non-linear encoding of the rest of the organisms genotype. Therefore, we investigated the evolution of assortativeness also under an alternative implementation, in which the level of assortativeness was coded for by the expression states of 6 extra assortativeness genes that lie randomly distributed amidst the other genes of the genome. We take the binary expression pattern of these 6 genes to code for the level of assortativeness in a non-linear non equivalent fashion (000000 gives assortativeness -1, 111111 gives assortativeness 1, 111000 gives assortativeness -0.7778, whereas 000111 gives assortativeness 0.7778). Figure S9C shows the evolution of assortativeness in the default model and under these settings. We can see that if assortativeness is coded for by 6 separate genes, assortativeness evolves even more readily than in the situation where assortativeness is coded for by a single parameter. In addition, higher levels of assortativeness are reached.

In hindsight this is not surprising. First, by coding for it by 6 genes rather than a single parameter that evolves in small steps, mutational stepsizes have increased allowing evolution to proceed faster. Second, we already found that the evolution of different phenotype patterns adapted to different niches stretching over 20 genes is quite easy, even despite random mating. The evolution of a single phenotype pattern (all organisms, irrespective of niche, "want to" become assortative) stretching over 6 genes should therefore be expected to be even easier.
